# EPIPOI: A user-friendly analytical tool for the extraction and visualization of temporal parameters from epidemiological time series

**DOI:** 10.1186/1471-2458-12-982

**Published:** 2012-11-15

**Authors:** Wladimir J Alonso, Benjamin JJ McCormick

**Affiliations:** 1 Fogarty International Center, National Institutes of Health, Bethesda, 20892, MD, USA

**Keywords:** Epidemiology, Time series, Trends, Seasonality, Anomalies, Data visualization

## Abstract

**Background:**

There is an increasing need for processing and understanding relevant information generated by the systematic collection of public health data over time. However, the analysis of those time series usually requires advanced modeling techniques, which are not necessarily mastered by staff, technicians and researchers working on public health and epidemiology. Here a user-friendly tool, EPIPOI, is presented that facilitates the exploration and extraction of parameters describing trends, seasonality and anomalies that characterize epidemiological processes. It also enables the inspection of those parameters across geographic regions. Although the visual exploration and extraction of relevant parameters from time series data is crucial in epidemiological research, until now it had been largely restricted to specialists.

**Methods:**

EPIPOI is freely available software developed in Matlab (The Mathworks Inc) that runs both on PC and Mac computers. Its friendly interface guides users intuitively through useful comparative analyses including the comparison of spatial patterns in temporal parameters.

**Results:**

EPIPOI is able to handle complex analyses in an accessible way. A prototype has already been used to assist researchers in a variety of contexts from didactic use in public health workshops to the main analytical tool in published research.

**Conclusions:**

EPIPOI can assist public health officials and students to explore time series data using a broad range of sophisticated analytical and visualization tools. It also provides an analytical environment where even advanced users can benefit by enabling a higher degree of control over model assumptions, such as those associated with detecting disease outbreaks and pandemics.

## Background

Government, non-governmental agencies and research groups regularly monitor the health status of populations with diverse geographical and temporal resolutions in addition to varying levels of clinical detail (from mortality records to DNA sequencing of particular pathogens) [1]. This invaluable documentation has enabled the development of elucidative studies about the causes of disease, increasing the likelihood of appropriate decision-making for public health.

Epidemiological data is frequently indexed in space (the locality where the event took place) and time and therefore, if systematically collected, many insightful and relevant patterns, outliers and parameters can be uncovered. For examples of the utility of spatio-temporal analysis one might consider the identification of the mortality attributed to epidemics [2-5] or exploring the spatial patterning of disease burden [6-9]. Much of this information, however, tends to be hidden to all but specialists with access to complex analytical tools. This complexity not only hinders a broader base of public health workers to further understand their own data (which can be critical in the preparation of responses for epidemics), but also inhibits the crosschecking of analyses and assumptions by people who understand the biology though perhaps not the computational programs used.

Here a freely available program, called "EPIPOI" (Epidemiological Parameter Investigation from Population Observations Interface), is presented which facilitates the visual exploration and description of several time series properties in a user-friendly and interactive way. Prototypes of EPIPOI have already been used in several of our studies [5,7,10,11] and have benefitted from this iterative experience. The development of the software was initiated in the context of the Fogarty International Center’s project the Multinational Influenza Study of Mortality and Seasonality (MISMS, http://www.origem.info/misms), where it was used for the inspection of pneumonia and influenza datasets. Its accessibility and utility was trialed by participants of several MISMS workshops around the world. More recently, EPIPOI was extended to assist in the analysis of the other vital statistics datasets within The Etiology, Risk Factors, and Interactions of Enteric Infections and Malnutrition and the Consequences for Child Health (Mal-ED) project ( http://mal-ed.fnih.org).

A number of excellent resources can be found online that do similar tasks to EPIPOI, however none combine the various specialist tasks into a single interface or present results in a manner that might offer additional insights beyond the raw analytical results – of particular interest in EPIPOI is the comparison of time series parameters in geographic space. For example, Pelat *et al.*[12] provide an excellent tool for periodic regression with a particular focus on predicting and identifying epidemics and Torrence and Campo [13] have an online tool to perform wavelet analysis (which EPIPOI has benefited from). In both cases only a single time series can be analyzed at a time, and consequently users must know how to manipulate extracted results in such a way to allow further comparison (indeed even recognizing that further comparison across time series might be made). Perhaps surprisingly, the closest existing software– PAST [14] – was developed for paleontology. PAST is able to visualize, fit and extract seasonal harmonics, but falls short of attempting to visualize time series parameters in the context of other comparable time series.

What follows is a brief technical introduction and discussion of features of EPIPOI, with further practical instructions and a repository for the program available at http://www.epipoi.info.

### Overview of EPIPOI

**Figure 1:**
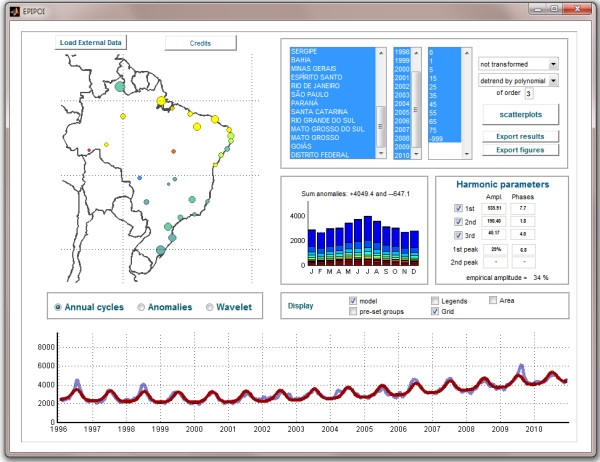
**Screenshot of EPIPOI showing pneumonia and influenza mortality data from Brazil (available as an example file on the EPIPOI website).** Circles in the map represent the amplitude (size) and timing of peaks (colours) of the seasonal signal of each Brazilian state as obtained with the default program settings. They were automatically calculated when the data were loaded and the detrending option was selected (2nd dropbox menu at upper-right corner). The model (red line over the original time series in grey) was obtained by summing the 12-monthly, 6-monthly and 3-monthly harmonics plus the trend. The central histogram indicates the age-profile of mortality for each month of an average year.

EPIPOI is meant to be self-explanatory. Users are guided to load their datasets and explore analytical options easily and with little worry of "doing something wrong". The dynamic appearance of options, display of meaningful keywords (technical jargon is avoided as much as possible), immediate visual feedback of the impact of options chosen (and therefore model assumptions) and consistent use of non-alphabetical coded information (e.g. colour keys) is intended to allow the user to concentrate on the exploration of their dataset (Figure 1).

## Implementation

EPIPOI has been written and compiled in Matlab (The Mathworks Inc) taking advantage of many built-in and second-party (including the authors) functions that are used to identify relevant information from time series. The immediate focus of our analyses is epidemiological, but nothing prevents users from other fields (e.g. ecology, economics) from finding EPIPOI analytically convenient and useful.

Figures displayed on the graphic user interface can, in the same way as numeric results, be generated at any stage of the analyses (and can be exported in several file formats).

### Preparation of data

EPIPOI accepts inputs from Microsoft Excel (because of its familiarity to most users) and then manipulates data internally to extract and export relevant parameters and processed data in formats ready to be exported back to Excel.

### Time series file

Users can load the example dataset provided on the EPIPOI website or use it as a template for their own data. The spreadsheet containing the data is organized in a simple structure: after a single header row, the first column is for the year, the second for a finer resolution time unit (e.g. months, weeks or days), and thereafter each column is dedicated to a series of observations (see the example on the EPIPOI website for an illustration). These time series need to cover the whole date range of years without any data gaps, though missing observations can be filled with NaN (the Matlab shorthand for “Not a Number”). All dates and observations must be sorted in ascending chronological order (i.e. oldest in the uppermost row and most recent at the bottom). If the data has an additional categorization (for instance age or gender), different worksheets of the time series file can be used to explore this "third dimension" of the data (time and -usually- space represent the other two dimensions). We encourage the user to explore this resource whenever possible (see the website for further details on how to do it).

### Geolocation file

In case each time series (column) of the "Time series file" corresponds to a different geographic location, a second file - the "geolocation file" - containing the latitudes and longitudes of each location is needed because EPIPOI can then display the temporal parameters of those series in geographic space as well. The geolocation file does not have headers, and only needs three columns: the names of the sites (which should be identical to the ones in the time series file) and then the latitudes and the longitudes (in decimal degrees). An optional forth column can be added with names of major groupings, such as country or province names as this additional information can be color coded in the maps and scatter plots.

## Results

### Overview of the analytical capabilities

Time series can be described by three components: a trend, their seasonality and the anomalies [4,15,16]. Each of these components can be of interest depending on the research question. Trend analyses inspect whether diseases have long term patterns, for example whether incidence is increasing or decreasing. Seasonal variation is useful for understanding how and when epidemics usually occur, and to provide insights on environmental or behavioral drivers of such patterns. Finally, anomalies can illustrate, for instance, morbidity and mortality figures in particularly severe epidemics (or pandemics) as compared to those figures expected based on the trend and regular seasonal and inter-annual variance in surrounding years [4,5].

EPIPOI also allows all parameters describing the components of time series from different locations to be plotted and analyzed spatially. For example, it can show that the timing of the annual peak of a disease changes along a latitudinal gradient [7], or radially from a certain point [11].

The interface is a pivotal part of the program, transforming a set of exploratory and analytical tasks - which would usually involve the use of several programs or the development of specific functions by specialists – into simple steps performed in few minutes by any user. Options are presented in a way such as to walk the user through basic steps before they can perform more complex analysis, thereby ensuring that they have both an appreciation of patterns within their data and are alerted to the increasing assumptions in more sophisticated analytical stages.

As soon as users load their time series, a plot of the data is presented (including a map, when appropriate) and several relevant parameters are automatically extracted. Those parameters can be compared across time series (see "scatterplot" button, figure 1) and exported (both numerically and graphically). Although the default input values can already provide users with insightful information, it is usually the case that further tuning the program’s options will provide even more valuable information. For instance, all data is used to build the default model, but it might be the case that the time series includes atypical years that should be excluded (not only to improve the description of typical years, but also to identify key features of irregular ones).

### Trend

Long-term changes in the average of a time series can be described in EPIPOI using a polynomial regression (the user can determine the polynomial order, with cubic as the default) (Figure 1). For instance, the coefficient of a linear trend term can be compared geographically to identify regions with an increase or decrease of a certain disease.

In some cases, the trend is of no analytical interest - for instance, when the research question concerns relative intra-annual seasonal dynamics so that detrending (i.e. subtracting the trend from the time series) can also be performed with a cubic spline [17], which does not output parameters but is a highly flexible option to describe complex long term patterns.

### Seasonality

**Figure 2:**
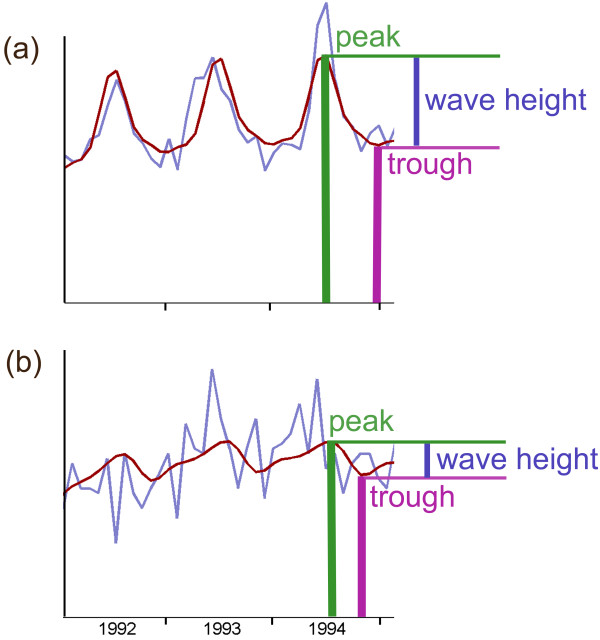
**Seasonal parameters obtained from mortality data of two different sites ("a" and "b").** The time series in light blue represents three years of raw data, whereas the overlapped red curve represents the model trend and seasonality. In the last year of the series the primary peak and trough of each series are highlighted. The amplitude of the periodic annual function is obtained by dividing the wave height (difference between the peak and trough values) by the peak value. Despite the troughs of both “**a**” and “**b**” being similar, the standardized wave is much bigger in "a" (making it easier to infer that the resulting amplitude will be higher in "**a**"). Note that series "**b**" has a secondary peak - i.e. is a bimodal periodic annual function (the calculation of the amplitude for this secondary peak follows the same logic as that used for the primary peak).

The seasonal parameters that EPIPOI extracts are the timing and magnitude of annual peaks in the time series. Those parameters indicate the moment when the maximum annual intensity of disease burden usually occurs, and how strong this cyclic variation is. EPIPOI describes this magnitude by dividing the wave height (difference between the peak and trough values) by the peak value to give an amplitude that is relative to the magnitude of the average seasonal signature of the original time series (Figure 2).

In the average seasonal signature of the original data year-to-year variations (trends and anomalies) are removed (or ignored, because they are negligible), but seasonal variations within the year are preserved. This seasonal signature could be obtained simply by interpolating the monthly (or weekly) averages of the time series. However, EPIPOI decomposes the time series into three sinusoids that can be related back to natural phenomena – 12-monthly, 6-monthly and 3-monthly cycles. These cycles make up a partial Fourier series (Figure 1, "harmonic parameters" box) [18,19], an approach that is not dissimilar to a periodic regression [20], but provides the trigonometric parameters as amplitude and phase rather than regression coefficients of sine and cosine functions. This approach enables the use of parameters of the individual harmonics themselves to quantify epidemics [4,21], or to identify putative, biologically relevant, descriptors of seasonality [18,22-24].

Finally, an important aspect of the investigation of the seasonal parameters is that the main period of analysis must be reasonably ‘stationary’ (i.e. no overall trends in the mean, the variance or the timing or peaks with time). If, for instance, the timings of the peaks in a later period are progressively shifted from an earlier period, users should split the series into smaller -and stationary - units and perform the analyses separately for each one of them.

### Anomalies

Once the trend and seasonality are described, anomalies or residuals must be considered. As mentioned, depending on the nature of the study they might be either the focus of analysis or ignored as noise. Examples of the former include determining excess mortality due to pandemic influenza compared to regular influenza seasons [5,25], or excess deaths of a disease that occurs in specific periods every year [26]. The latter approach has been used in influenza modeling to estimate baseline mortality using the trend and seasonal components of mortality series, but excluding those weeks or months that are known (from laboratory data) to encompass seasonal epidemics. The key assumption of this method is that variation from year to year in the disease of interest (in this case influenza) overshadows the inter-annual variation in the timing of occurrence of all remaining pathogens.

It is important to highlight that, of the analyses available in EPIPOI, the treatment of anomalies is certainly the most sensitive to choices made by the user (such as the exclusion of specific time periods), hence awareness about the assumptions made and their limitations is required. This is not something particular to EPIPOI. By definition, the identification of departures from a model will depend crucially on the model formulation itself. It is worth noting that the goodness of fit of a model is based on the magnitude of anomalies, so anomalies are minimized in the modeling process.

**Figure 3:**
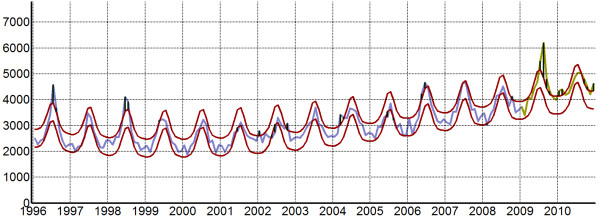
**Image exported from EPIPOI showing the exclusion of two years from the data (2009–2010, highlighted in yellow).** The 95% confidence intervals of the model are shown in addition to the fitted model. Observations exceeding these limits (e.g. January and February 2002), suggest epidemics that would not be expected based on the seasonal and overall trend patterns (e.g., the 2009 influenza pandemic, [5]).

That said data exclusion is an important tool in modeling. This can be illustrated for a pandemic scenario: after careful inspection of the data, observations within the 95% prediction interval of the time series might be considered (by convention) as regular variability. If, however, we consider a time series that contains one year with a particularly severe epidemic (e.g. a pandemic), accounting for the trend, seasonality and variability of the entire time series may bias the model as the fit will account for the unusually high pandemic disease burden. In order to estimate the excess mortality attributable to the pandemic, we need first to eliminate the pandemic year to build a model that represents baseline burden in the absence of a pandemic (Figure 3). Mortality above the prediction interval (i.e. expected disease burden variability for non-pandemic years) then represents the anomalous mortality during the pandemic period [5].

Alternatively, anomalies might be considered biased observations or one-off outbreaks. In such cases, data can be optionally smoothed using digital or moving average filters (from the "transformation” drop-down menu).

### Wavelets

EPIPOI also makes wavelet analysis available (courtesy of code from Torrence and Compo [27]). Wavelet analysis is a sophisticated technique that describes the power of sinusoidal patterns of a time series with different frequencies (like the 1-year, 6-month and 3-month harmonics of the seasonal analysis, but including a continuum of other periodicities). In EPIPOI, its main utility is to determine how those periodicities might be changing over time. For instance, shifts from a periodicity of predominantly one year to two years, resulting perhaps from an intervention [9] or from changing climatic drivers [28] – might be detected.

### Visualizing data

**Figure 4:**
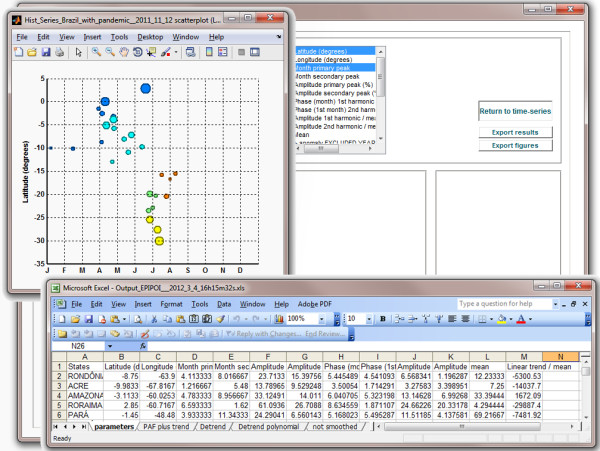
**Figure and Excel file exported from EPIPOI.** The graph shows a latitudinal gradient in the timing of peak pneumonia and influenza mortality in Brazil, illustrating the utility of comparing the seasonality of time series geographically [7]. Like in the map in Figure 1, EPIPOI plots the size of markers based on selected parameters, though in this case the color denotes the regions of Brazil. The identity of each observation can be optionally added to the scatter plot using the ‘legends’ check-box. EPIPOI can also export all parameters extracted into a file ready for further analysis. All parameters are available for each location.

A central feature of EPIPOI is the facility to plot the time series parameters extracted (scatterplot option). With several time series, comparing these parameters can be insightful, particularly for geocoded series with unique latitude and longitude values. With geographic information, parameters can be visualized as maps (e.g. Figure 1) where means, amplitudes or timing of peaks can be plotted against latitudes or longitudes for the identification of spatial trends (Figure 4) [7,10,11].

If the scatterplot option is chosen in the anomalies mode, the timing and magnitude of anomalies (both above and below the optional prediction interval, and from either the excluded periods or the whole series) are available for inspection on the plot of the time series.

**Figure 5:**
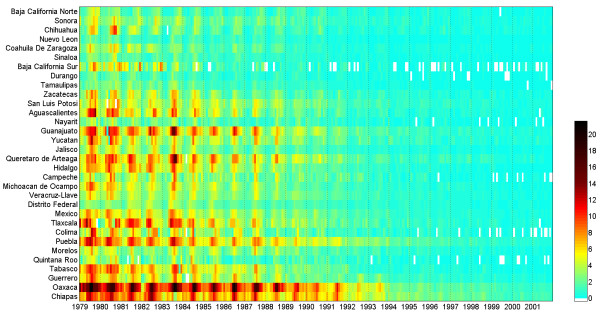
**Heat grid graphic exported from EPIPOI, showing incidence mortality of diarrhea in a latitudinal gradient across Mexico**[11]. Heat grids are a remarkable way of visualizing numerical matrices because heat grids are, in fact, matrices with the particularity that values are expressed in a gradient of tones or colors, instead of numbers. In this heat map, it is possible to identify differences in trends, anomalies and peak times (even a switch from summer to winter peaks) without the use of any calculation.

A map containing markers whose sizes are proportional to the amplitudes (and colors representing the monthly timing of peaks) of their time series is provided by default from the moment the user loads the time series data. However, an alternative visualization is a matrix of the time series rather than a map. In this heat grid (figure 5) data are visualized with time along the horizontal axis (columns) and the time series ordered by latitude (if supplied or the order of columns if not) (rows). Results are then shown using a colour ramp, which can be more striking than examining raw numbers. This visualization can reveal patterns of synchrony in the timing of peaks across the different time series and whether such synchrony dissolves due to diverging driving forces (e.g., differential patterns of socio-economic growth [11]). When time series do not represent data collected from different sites, but relate to other features, like age groups or different pathogens, the inspection of their properties both by scatterplots and heat grids can also be insightful, particularly if the columns of data are ordered in a meaningful way in the original spreadsheet (e.g. age-groups in ascending order, or pathogens that caused each diseases grouped by taxonomic similarity). It is also worth noting that users can export the figures displayed on the screen at any stage (and in several formats).

## Results and discussion

Reproducibility of results has been advocated as a fundamental pillar of the scientific method [29]. With EPIPOI this possibility is taken to its full meaning for epidemiologic time analyses because any user can not only reproduce (and even improve) the analyses we have previous performed, but also apply, compare and test the same methods with other datasets. In so doing, EPIPOI expands the number of users that can perform these analyses and can be instrumental in helping process epidemiological data currently available yet still unexplored in many countries.

EPIPOI packages a number of useful resources for the exploration and visualization of temporal patterns that ought to be part of a first step in any analysis [30], but are largely confined to those with knowledge of specialist software.

This ease of exploring parameters resulting even from complex analysis warrants, on the other hand, constant awareness of the various transformations and assumptions introduced in the analytical process (as detrending, or exclusion of periods from the model). This latter feature is a methodological advantage over approaches that rely simply on goodness of fit. In EPIPOI the user can, for instance, identify the tension or bias that data from a particular period produces in the model, leading to a refinement of the analyses (e.g. was a particular pathogen or strain circulating at that time? is this phenomena structured geographically? were all age groups affected similarly?).

EPIPOI does not, however, cover all analytical possibilities that can be of relevance in the investigation of epidemiological time series. For instance, EPIPOI does not calculate the reproductive number of a pathogen, nor does it currently allow the inclusion of additional variables (e.g., climatologic or other putative risk factors) in the model. Some of these limitations are restricted to the current version, and will be addressed in future releases. But others stem from our reluctance to interfere with the simplicity and friendliness of the user experience. Finally, there are also analytical possibilities that we did not include for epistemological reasons. Chiefly, we have been frequently asked why EPIPOI does not make predictions. Although it would be possible to include an option to extrapolate model results, we believe EPIPOI is a tool to better explore and understand relevant descriptive information in empirical data. Error rates grow very rapidly as models project into the future and new conditions can suddenly change the premise upon which they were built [31] such that public health decisions should be based on evidence and insight, rather than reliant solely on on models (especially when their underpinning assumptions and limitations are difficult to check and/or understand).

Overall, EPIPOI has already proved useful to public health practitioners (trialed at MISMS workshops around the globe), contributing to several research studies [5,7,10,11] emphasizing a need and desire for wider access to such a tool. We believe it can be used on a larger scale and perhaps in fields not originally anticipated. Feedback from users and the constant need to stretch analytical possibilities should encourage the development of new versions.

## Conclusions

The accessibility of free and user-friendly tools for exploratory analysis of spatio-temporal data, including data visualization, has been limited. It is hoped that EPIPOI can address this imbalance and make available hitherto specialist tools in an intuitive format, so that a wider array of public health practitioners can explore their own epidemiological time series.

## Availability and requirements of the current version (1.0)

Project name: EPIPOI

Project home page: http://www.epipoi.info

Operating system: 64-bit Windows PC and Macs (10.6.4 and above)

Programming language: Matlab (The Mathworks Inc)

Other requirements: Matlab or the Matlab Compiler Runtime (MCR). The EPIPOI software, the link to the MCR and further instructions and learning material can be found at the EPIPOI website

License: GNU

Any restrictions to use by non-academics: None, provided that - as is the case with academics - the proper citation to the current article (and when relevant, to the website) is provided.

## Competing interests

The authors declare that they have no competing interests.

## Authors’ contributions

AJW wrote the original basis of EPIPOI with contributions from BJJM on specific functions. Both authors wrote, read and approved the final manuscript.

## Pre-publication history

The pre-publication history for this paper can be accessed here:

http://www.biomedcentral.com/1471-2458/12/982/prepub
